# Combining Functional and Structural Genomics to Sample the Essential *Burkholderia* Structome

**DOI:** 10.1371/journal.pone.0053851

**Published:** 2013-01-31

**Authors:** Loren Baugh, Larry A. Gallagher, Rapatbhorn Patrapuvich, Matthew C. Clifton, Anna S. Gardberg, Thomas E. Edwards, Brianna Armour, Darren W. Begley, Shellie H. Dieterich, David M. Dranow, Jan Abendroth, James W. Fairman, David Fox, Bart L. Staker, Isabelle Phan, Angela Gillespie, Ryan Choi, Steve Nakazawa-Hewitt, Mary Trang Nguyen, Alberto Napuli, Lynn Barrett, Garry W. Buchko, Robin Stacy, Peter J. Myler, Lance J. Stewart, Colin Manoil, Wesley C. Van Voorhis

**Affiliations:** 1 Seattle Structural Genomics Center for Infectious Disease, Seattle, Washington, United States of America; 2 Department of Medicine, Division of Allergy and Infectious Disease, University of Washington, Seattle, Washington, United States of America; 3 Department of Genome Sciences, University of Washington, Seattle, Washington, United States of America; 4 Emerald BioStructures, Bainbridge Island, Washington, United States of America; 5 Seattle Biomedical Research Institute, Seattle, Washington, United States of America; 6 Biological Sciences Division, Pacific Northwest National Laboratory, Richland, Washington, United States of America; 7 Department of Global Health, University of Washington, Seattle, Washington, United States of America; 8 Department of Microbiology, University of Washington, Seattle, Washington, United States of America; 9 Department of Medical Education and Biomedical Informatics, University of Washington, Seattle, Washington; University of Florida, United States of America

## Abstract

**Background:**

The genus *Burkholderia* includes pathogenic gram-negative bacteria that cause melioidosis, glanders, and pulmonary infections of patients with cancer and cystic fibrosis. Drug resistance has made development of new antimicrobials critical. Many approaches to discovering new antimicrobials, such as structure-based drug design and whole cell phenotypic screens followed by lead refinement, require high-resolution structures of proteins essential to the parasite.

**Methodology/Principal Findings:**

We experimentally identified 406 putative essential genes in *B. thailandensis*, a low-virulence species phylogenetically similar to *B. pseudomallei*, the causative agent of melioidosis, using saturation-level transposon mutagenesis and next-generation sequencing (Tn-seq). We selected 315 protein products of these genes based on structure-determination criteria, such as excluding very large and/or integral membrane proteins, and entered them into the Seattle Structural Genomics Center for Infection Disease (SSGCID) structure determination pipeline. To maximize structural coverage of these targets, we applied an “ortholog rescue” strategy for those producing insoluble or difficult to crystallize proteins, resulting in the addition of 387 orthologs (or paralogs) from seven other *Burkholderia* species into the SSGCID pipeline. This structural genomics approach yielded structures from 31 putative essential targets from B. thailandensis, and 25 orthologs from other *Burkholderia* species, yielding an overall structural coverage for 49 of the 406 essential gene families, with a total of 88 depositions into the Protein Data Bank. Of these, 25 proteins have properties of a potential antimicrobial drug target i.e., no close human homolog, part of an essential metabolic pathway, and a deep binding pocket. We describe the structures of several potential drug targets in detail.

**Conclusions/Significance:**

This collection of structures, solubility and experimental essentiality data provides a resource for development of drugs against infections and diseases caused by *Burkholderia*. All expression clones and proteins created in this study are freely available by request.

## Introduction

Gram-negative bacteria of the genus *Burkholderia* include the pathogenic species *B. pseudomallei* and *B. mallei*, potential bioterrorism agents and the causative agents of melioidosis and glanders, respectively, and *B. cenocepacia*, which causes often-fatal pulmonary infections in patients with cancer and cystic fibrosis [1]–[3]. Treatment of these infections is challenging due to intrinsic and acquired drug resistance [4], [5]. New approaches are needed to develop antibiotics less susceptible to drug resistance.

A first step in focusing a search for new antimicrobials is to identify the set of genes required for survival of the pathogen. Methods to determine a minimum set of essential genes include experimental approaches based on genome-wide gene disruption or systematic mutagenesis [6]–[10], and bioinformatic methods based on comparative analysis of genomes [11], [12]. Experimentally determined counts of essential genes in infectious bacteria range from <200 to >600 [10]–[13], with estimates for *Burkholderia* using computational methods ranging from 312 to 649 [11], [14]. There have been no whole-genome essentiality studies in the genus *Burkholderia*. The order *Burkholderiales* was estimated to have 610 orthologous gene families conserved among all 51 species, using an all-against-all BLAST search of the 51 proteomes and clustering into ortholog groups using OrthoMCL [11]. These 610 ortholog groups corresponded to 649 genes in *B. cenocepacia*, 454 of which had homologs in the Database of Essential Genes (DEG) [15]. In *B. pseudomallei*, 312 putative essential genes that lack close human homologs were predicted based on comparison of the *B. pseudomallei* proteome with the DEG and with the human proteome [14]. A set of 335 putative essential genes was identified experimentally in *P. aeruginosa*, a pathogen phylogenetically similar to *B. cenocepacia*, using saturation-level transposon mutagenesis [16], while a different study of *P. aeruginosa* also using saturation transposon mutagenesis estimated 300–400 essential genes [17].

Together with knowledge of essential functions, another critical resource for developing new antimicrobials is a set of high-resolution three-dimensional structures for the corresponding proteins. Such structures are required for structure-guided drug lead design and refinement. Improvements in high-throughput protein expression and structure determination methods have improved the overall gene-to-structure success rate, but this rate typically remains relatively low (<10%) due to insolubility of a high percentage of proteins in heterologous expression systems [18], [19], and intractability of other proteins to crystallization or structure determination. One strategy that has been employed to improve success rates is to “rescue” such proteins by adding orthologs from related species to the pipeline [18], based on the assumptions that many of these will have slightly different physical properties that may improve their solubility or crystallization, and that close orthologs will have structures sufficiently similar to the original target to be useful as surrogates in drug design [18], [20], [21].

In this study, we apply saturation-level transposon mutagenesis and next-generation sequencing (Tn-seq) to identify putative essential genes in *B. thailandensis*, a low-virulence species with a genome closely related to that of *B. pseudomallei* [22] and sharing numerous physiologic and virulence traits [23]–[25]. We then applied high-throughput structure determination with an “ortholog rescue” approach to maximize structural coverage of these essential genes. For each essential gene product with a structure solved, we analyze the protein for properties of a potential antibacterial drug target, such as lacking a close human homolog, being a member of an essential metabolic pathway (having ≥2 essential enzymes), and possessing a binding pocket capable of enveloping a compound of at least six non-hydrogen atoms. We describe five of these potential drug targets in detail. The resulting collection of structures and information about target essentiality and solubility provides a resource for development of new antibiotics to treat *Burkholderia*-related infectious diseases.

## Results

### Experimental Determination of Putative Essential Genes in *B. thailandensis*

The genome of *B. thailandensis* E264 consists of 6.72 million base pairs and 5712 predicted genes. We used saturation-level transposon mutagenesis followed by next-generation sequencing to identify putative essential genes (see Materials and Methods). Two independent pools of mutants were generated with >30 insertions per gene, and insertion locations were identified by Tn-seq, a technique which uses next-generation sequencing to profile complex pools of insertion mutants [26]. Genes with no, or only a few (<10% of the average per gene density), insertions in both pools were considered putative essential genes. A total of 406 such genes were identified, representing 7.1% of the total predicted gene set of *B. thailandensis.* These results are summarized in Table 1; the complete set of putative essential genes with number of insertions per kB is listed in Table S1.

**Table 1:** Identification of essential genes using saturation transposon mutagenesis and Tn-seq.

	Pool 1	Pool 2
Approximate number of mutants pooled	170,000	220,000
Insertion locations (hits) identified	171,719	218,928
Hits within coding genes	144,503	182,951
Genes without insertions in 5th–90th percentileof ORF	398	346
Genes with <3 insertions/kB in 5th–90th percentile of ORF	538	453
Putative essential genes (<3 insertions/kB in both pools)	406	

We examined these genes by mapping them to metabolic pathways using the Kyoto Encyclopedia of Genes and Genomes (KEGG) [27], and by comparing them with genes previously identified as essential in related organisms (Table S1). Prior to this study, there had been no experimental genome-wide essentiality studies in *Burkholderia*. We searched for homologs among genes predicted to be essential in *B. cenocepacia* (from the computationally defined “core genome” in *Burkholderiales*, based on gene conservation among all 51 species in the order with an available genome sequence [11]); in *P. aeruginosa* (based on saturation transposon mutagenesis [16]); and in the Database of Essential Genes (DEG), a collection that includes 7430 prokaryotic genes [15]. We used a BlastP search with an *E*-value cutoff of 1×10^−10^ and a minimum 30% sequence identity over at least 50% of the sequence to identify homologs. Of our 406 putative essential genes, 349 (83%) had homologs identified as essential in other bacteria; 241 of 406 had homologs among the core genome of *B. cenocepacia*; 330 of 406 had homologs in the DEG (including all but three of the 241 *B. cenocepacia* homologs), and we found 13 additional homologs among genes identified as essential in *P. aeruginosa* [16]. Table S1 lists the closest homologs (best hits) and their percent sequence identity. We found no homologs for 70 of the 406∶48 of these have homologs in the *B. cenocepacia* proteome not in the “core genome” [11], while 27 were annotated “protein of unknown function.”

We also used BlastP to map 265 of the 406 *B. thailandensis* genes onto 62 different KEGG [27] metabolic pathways (see Table S1). Several pathways essential for bacterial growth (such as the histidine, purine, and pyrimidine biosynthetic pathways, tRNA charging pathways, and the aspartate pathway) were over-represented, despite the use of rich growth medium containing amino acids and nucleosides. Other pathways that are thought to be non-essential for *in vitro* growth (including those for aromatic compound degradation and UDP sugar interconversion) were under-represented.

### Selection of Targets for Expression and Structure-determination

The 406 *B. thailandensis* putative essential gene targets were processed according to normal SSGCID target selection criteria: eliminating proteins with over 750 amino acids, 10 cysteines, or 95% sequence identity with 70% coverage to proteins already in the PDB, targets being worked on by other groups, and targets with transmembrane domains (except where a soluble domain could be expressed separately). Using these criteria, 315 of the 406 *B. thailandensis* essential genes were selected for cloning.

Since we expected a modest success rate for these 315 targets, we also implemented an “ortholog rescue” strategy to increase the likelihood of solving a structure for each gene product. Orthologs (and paralogs) of the 315 *B. thailandensis* genes were identified in seven other *Burkholderia* species (*B. pseudomallei*, *B. cenocepacia, B. ambifaria*, *B. multivorans*, *B. phymatum*, and *B. xenovorans*) selected based on their medical significance and phylogenetic diversity (we sought to maximize the coverage of sequence space). To identify orthologs, we used a BlastP search of the 315 selected *B. thailandensis* genes against the proteomes of these species using a cutoff of 40% sequence identity over 70% of the sequence, and clustered the resulting sequences into ortholog groups using OrthoMCL [28], [29]. These “ortholog groups” include both orthologs and in-paralogs – we will use “orthologs” to include both. Based on this search, an additional 387 orthologs from these seven *Burkholderia* species were selected, bringing the total number of targets selected for structure determination to 702.

### High-throughput Structure Determination

Target progress by *Burkholderia* species as of October 1, 2012 is shown in Table 2. We stopped work on any target for which an ortholog structure was solved, except in five cases in which multiple orthologs were too far along in the structure determination process to warrant not completing deposition into the PDB. Out of 702 targets approved by the NIAID, 698 were selected for cloning and 675 were successfully cloned from genomic DNA. In small-scale screening, 450 of the 675 cloned targets (67%) showed soluble expression with an N-terminal His_6_-tag. Of these 450 soluble proteins, 170 crystallized (38%) and 68 proteins diffracted with sufficient resolution to meet SSGCID quality criteria and were submitted to the PDB. A total of 88 structures were deposited into the PDB, including ligand-bound structures. X-ray crystallography data are summarized in Table S3. As shown in Table 3 (and in the expanded version, Table S2), structures were solved for 31 *B. thailandensis* targets and 25 targets in other *Burkholderia* species –56 total *Burkholderia* proteins – representing 49 *B. thailandensis* putative essential genes.

**Table 2:** Target progress by *Burkholderia* species.

	*thailand-ensis*	*pseudo-mallei*	*ceno-cepacia*	*ambi-faria*	*phyma-tum*	*vietnam-iensis*	*xeno-vorans*	*multi-vorans*	Total
Target approved	315	23	57	57	64	67	68	51	702
Selected	315	23	54	57	64	67	67	51	698
Cloned	302	23	52	57	61	64	66	50	675
Expressed	260	23	52	45	55	61	63	41	600
Soluble	226	22	32	30	38	41	30	31	450
Purified	134	21	18	18	24	20	17	23	275
Crystallized	76	21	9	13	15	11	12	13	170
Diffraction	38	16	5	7	8	8	8	8	98
Native diffraction data	36	15	4	7	7	8	8	7	92
In PDB	31	14	1	4	2	1	3	0	56
Work stopped	5	1	3	3	5	7	5	6	35

**Table 3:** *Burkholderia* protein structures.

Protein with structure(s) solved	Protein name	PDB ID(s)	*Burkholderia* species	Putative essential gene	Human homolog*	% seq ID, coverage*	Deep pocket
BURPS1710b_0395	S-adenosylmethionine synthetase	3IML	*B. pseudomallei*	BTH_I0174	Q00266	58.7, 94.7	Yes
BTH_I0211	Ferredoxin-NADP reductase	4F7D, 4FK8	*B. thailandensis*	BTH_I0211	–	–	Yes
BTH_I0291	Dihydroneopterin aldolase	3V9O	*B. thailandensis*	BTH_I0291	–	–	Yes
BTH_I0294	Uncharacterized ACR	4F3N, 4G67	*B. thailandensis*	BTH_I0294	Q7L592	26.6, 92.9	Yes
BURPS1710b_0748	Phosphopantetheine adenylyltransferase	3K9W, 3PXU	*B. pseudomallei*	BTH_I0469	–	–	Yes
**BTH_I0472**	**Peptidyl-tRNA hydrolase (PTH)**	**3V2I**	***B. thailandensis***	**BTH_I0472**	**Q86Y79**	**36.4, 86.6**	**Yes**
BURPS1710b_0753	Ribose-phosphate pyrophosphokinase (prsA)	3DAH	*B. pseudomallei*	BTH_I0474	P11908	47.5, 98.1	Yes
BTH_I0484	PTS IIA-like nitrogen-regulatory protein PtsN	3URR	*B. thailandensis*	BTH_I0484	–	–	Yes
BTH_I0732	Ornithine carbamoyltransferase	4F2G	*B. thailandensis*	BTH_I0732	P00480	39.8, 96.1	Yes
BURPS1710b_1080	Adenylate kinase (adk)	3GMT	*B. pseudomallei*	BTH_I0739	P54819	48.5, 98.1	Yes
BURPS1710b_1108	Isocitrate dehydrogenase (icd)	3DMS	*B. pseudomallei*	BTH_I0759	P50213	31.4, 94.0	Yes
BTH_I0848	Pantothenate synthetase (panC)	3UK2	*B. thailandensis*	BTH_I0848	–	–	Yes
BTH_I0860	Deoxycytidine triphosphate deaminase (dcd)	4DHK	*B. thailandensis*	BTH_I0860	–	–	No
BURPS1710b_1237	Inorganic pyrophosphatase	6 structures	*B. pseudomallei*	BTH_I0878	–	–	Yes
BTH_I0882	Glutamine dependent NAD+ synthetase	4F4H	*B. thailandensis*	BTH_I0882	–	–	Yes
BTH_I1058	Triosephosphate isomerase	4G1K	*B. thailandensis*	BTH_I1058	P60174	41.5, 99.2	Yes
BamMC406_0490	D-alanine–D-alanine ligase (ddl)	4EG0	*B. ambifaria*	BTH_I1120	–	–	Yes
Bxe_A0488		4EGJ	*B. xenovorans*			–	Yes
BTH_I1195	Transketolase (tkt)	3UK1, 3UPT	*B. thailandensis*	BTH_I1195	Q53EM5	27.8, 95.8	Yes
BTH_I1208	Dihydrodipicolinate reductase	4F3Y	*B. thailandensis*	BTH_I1208	–	–	No
BTH_I1214	Gamma-glutamyl phosphate reductase	4GHK	*B. thailandensis*	BTH_I1214	P54886	37.8, 96.2	Yes
BTH_I1311	3-methyl-2-oxobutanoate hydroxymethyltransferase (panB)	3VAV	*B. thailandensis*	BTH_I1311	–	–	Yes
BTH_I1489	Phosphoglucomutase	3UW2	*B. thailandensis*	BTH_I1489	–	–	Yes
Bphy_0771	tRNA (guanine-N(1)-)-methyltransferase	4H3Z, 4H3Y	*B. phymatum*	BTH_I1663	O75588	84.6, 53.3	Yes
**BTH_I1680**	**Thymidylate synthase (thyA)**	**3V8H**	***B. thailandensis***	**BTH_I1680**	**Q53Y97**	**34.6, 99.4**	**Yes**
**BURPS1710b_0096**	**3-oxoacyl-ACP synthase III (FabH)**	**3GWA, 3GWE**	***B. pseudomallei***	**BTH_I1717**	**–**	**–**	**Yes**
**Bxe_A0096**		**4EFI**	***B. xenovorans***				**Yes**
**Bxe_A1072**		**4DFE**	***B. xenovorans***				**Yes**
BURPS1710b_2906	Acyl-carrier-protein S-malonyltransferase	3EZO	*B. pseudomallei*	BTH_I1718	Q8IVS2	32.6, 91.3	Yes
BURPS1710b_2905	3-ketoacyl-ACP reductase (fabG)	3FTP	*B. pseudomallei*	BTH_I1719	Q92506	43.2, 99.6	Yes
BURPS1710b_A1014	Acetoacetyl-CoA reductase	3GK3	*B. pseudomallei*			37.2, 97.6	Yes
Bcep1808_4002	3-oxoacyl-ACP synthase II	4DDO, 4F32	*B.vietnamiensis*	BTH_I1721	Q9NWU1	45.6, 96.1	Yes
Bphy_0703	Beta-ketoacyl synthase	4EWG	*B. phymatum*			34.7, 98.8	Yes
BURPS1710b_2892	Pyridoxal phosphate biosynthetic protein	3GK0	*B. pseudomallei*	BTH_I1733	–	–	Yes
BTH_I1883	Lysyl-tRNA synthetase (lysS)	4EX5	*B. thailandensis*	BTH_I1883	Q15046	39.3, 97.8	Yes
***BamMC406_2018***	***2-dehydro-3-deoxyphospho-octonate aldolase (kdsA)***	**3T4C**	***B. ambifaria***	**BTH_I1893**	**–**	**–**	**Yes**
**BCAL2180**		**3TML**	***B. cenocepacia***				**Yes**
**BURPS1710b_3264**		**3SZ8, 3TMQ, 3UND**	***B. pseudomallei***				**Yes**
BURPS1710b_2636	Enoyl-ACP reductase (fabI)	3EK2	*B. pseudomallei*	BTH_I1977	–	–	Yes
BTH_I1984	Glutamyl-tRNA synthetase (gltX)	4G6Z	*B. thailandensis*	BTH_I1984	Q5JPH6	35.5, 67.0	Yes
BTH_I2038	(3R)-hydroxymyristoyl-ACP dehydratase	4H4G	*B. thailandensis*	BTH_I2038	–	–	Yes
BTH_I2039	UDP-N-acetylglucosamine O-acyltransferase	4EQY	*B. thailandensis*	BTH_I2039	–	–	Yes
BamMC406_4587	Putative signal-transduction protein with CBS domains	4FRY	*B. ambifaria*	BTH_I2056	–	–	Yes
BURPS1710b_2511#	2C-methyl-D-erythritol 2,4-cyclodiphosphate synthase	17 structures#	*B. pseudomallei*	BTH_I2090	–	–	Yes
BTH_I2154	Thymidylate kinase (tmk)	3V9P	*B. thailandensis*	BTH_I2154	–	–	Yes
BTH_I2199	Threonine synthase (thrC)	3V7N	*B. thailandensis*	BTH_I2199	Q8N9J5	32.6, 88.4	Yes
BTH_I2231	Nucleoside diphosphate kinase	4DUT, 4EK2	*B. thailandensis*	BTH_I2231	Q9NUF9	44.7, 93.6	Yes
BTH_I2235	Histidyl-tRNA synthetase (hisS)	4E51	*B. thailandensis*	BTH_I2235	-	-	Yes
BTH_I2245	Adenylosuccinate synthetase	3UE9	*B. thailandensis*	BTH_I2245	Q8N142	42.0, 96.9	Yes
BTH_I2516	Ribose-5-phosphate isomerase A	3U7J, 3UW1	*B. thailandensis*	BTH_I2516	P49247	36.2, 95.3	Yes
BTH_I3037	GTP-binding protein engB	4DHE	*B. thailandensis*	BTH_I3037	Q8N3Z3	30.8, 71.7	Yes
BTH_I3304	Uroporphyrinogen decarboxylase	4EXQ	*B. thailandensis*	BTH_I3304	P06132	48.5, 98.1	Yes
BTH_II0675	Aspartate-semialdehyde dehydrogenase	3UW3	*B. thailandensis*	BTH_II0675	–	–	Yes
BamMC406_2543	Phosphoribosylaminoimidazole carboxylase, ATPase subunit	4E4T	*B. ambifaria*	BTH_II0682	–	–	Yes
BTH_II1941	Agmatinase, putative	4DZ4	*B. thailandensis*	BTH_II1941	Q9BSE5	42.5, 88.8	Yes
**BTH_II2229**	**Isochorismatase family protein**	**3TXY**	***B. thailandensis***	**BTH_II2229**	**–**	**–**	**Yes**

An expanded version of this table is available in the Supporting Information (Table S2).

Structures described in detail in the manuscript are indicated in bold.

* Best hit (if any) in a BlastP search against the human proteome, using an *E*-value cutoff of 1×10^−10^ (UniProtKB AC).

# BURPS1710b_2511 was screened using a fragment-based approach, yielding 17 PDB structures and 16 unique ligand-bound complexes: 3F0D, 3F0E, 3F0F, 3F0G, 3IEQ, 3IEW, 3MBM, 3P0Z, 3P10, 3Q8H, 3QHD, 3IKE, 3IKF, 3JVH, 3K14, 3K2X, 3KE1.

### Analysis of Solved Targets

We analyzed each of the 56 proteins for properties of a potential antimicrobial drug target: having no close human homologs (based on a BlastP search against the human proteome, using an *E*-value cutoff of 1×10^−10^ with >30% sequence identity and 50% coverage) (30/56), being a member of an essential metabolic pathway (having at least two enzymes with homologs in the Database of Essential Genes) (48/56), and possessing a binding pocket capable of enveloping a compound of at least six non-hydrogen atoms (54/56). The closest human homologs (best hits) are shown along with percentage sequence identity and coverage in Table 3. We used KEGG to identify one or more pathways for each protein (Table S2). To determine whether these pathways contained more than one essential enzyme, we obtained a list of all enzymes in each pathway from the KEGG, and performed a BlastP search of the sequences of these enzymes against the DEG (using an *E*-value cutoff of 1×10^−10^ and minimum 30% sequence identity and 50% coverage). Of the 56 proteins, 48 had a pathway listed in the KEGG, and all of these pathways had at least two enzymes with homologs in the DEG (Table S2). Of the 56 *Burkholderia* proteins with a structure solved, 25 satisfied all three criteria of a potential antimicrobial drug target listed above.

In five cases, we obtained structures from two or more orthologs of the same essential gene, although none of these cases included the original *B. thailandensis* target. To assess the structural similarity of orthologs, we calculated overall Cα RMSD values for all seven pairs of ortholog structures (without bound ligand) (Table S4). While these ortholog pairs had a mean amino acid sequence identity of 55±26% (1 standard deviation) with a mean coverage of 97%, in some cases the sequence identity was only 30–38%. Nevertheless, all ortholog pairs showed a high degree of structural similarity, with an average RMSD of 1.5±0.5 Å over all common Cα atoms. In general, pairs with greater sequence identity showed more structural similarity, but there were exceptions. For instance, while BURPS1710b_3264 showed 50% sequence identity to both BamMC406_2018 and BuceA.00102.a, the RMSD was 2.1 Å for the former, but only 1.4 Å for the latter. In contrast, Bxe_A1072 and Bxe_A0096 both showed an RMSD of 1.8 Å from BURPS1710b_0096, but had sequence identities of 30% and 38%, respectively.

### Structures of *Burkholderia* Putative Essential Proteins

**Figure 1:**
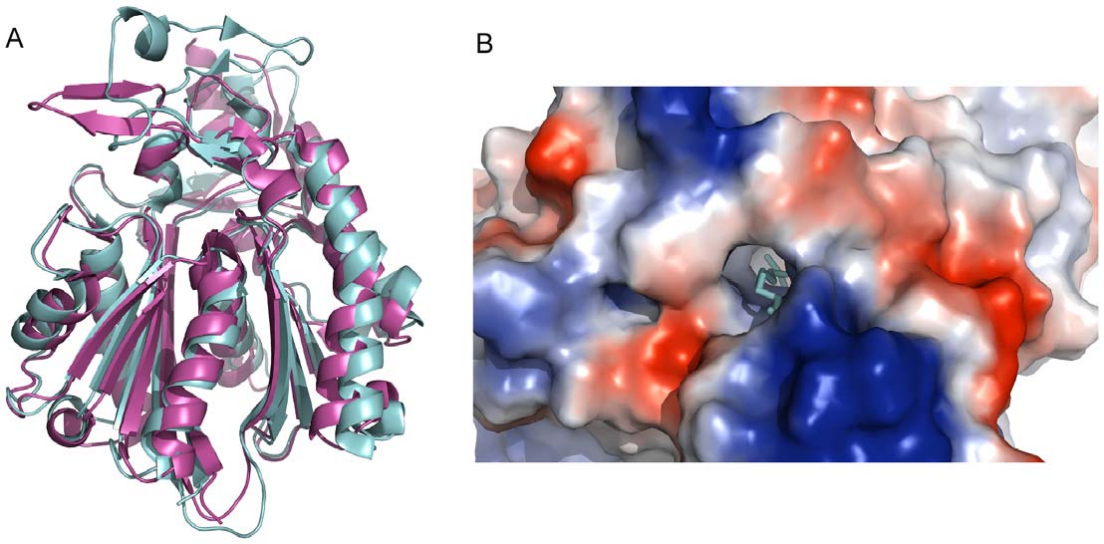
FabH structures from *B. pseudomallei* and B. xenovorans. (A) FabH (3-oxoacyl-(acyl-carrier-protein) synthase III) from *B. pseudomallei 1710b* (BURPS1710b_0096, PDB: 3GWA, cyan) and *B. xenovorans LB400 B* (Bxe_A1072, PDB: 4DFE, magenta) have similar overall structures, with a Cα RMSD of 1.8 Å between individual chains of 3GWA and 4DFE. There is no close human homolog based on a BlastP search of the human proteome. (B) In 4DFE, a hydrophobic tunnel to the active site is adjacent to a positively-charged surface patch (marked in blue).

FabH, which encodes 3-oxoacyl-(acyl-carrier-protein) synthase, is essential in the absence of long chain fatty acids in some species, such as *E. coli*, but not in others, such as *Pseudomonas aeruginosa* [30], [31], and has been identified as a promising drug target in pathogenic bacteria [32]. The *B. thailandensis* FabH gene (BTH_I1717) was among the group of genes we identified as essential for *in vitro* growth using rich medium. We solved structures for orthologs/in-paralogs of this gene in *B. pseudomallei* (BURPS1710b_0096, PDB: 3GWA and 3GWE) and *B. xenovorans* (Bxe_A0096, PDB: 4EFI and Bxe_A1072, PDB: 4DFE) (Figure 1). As discussed above, these structures are very similar, with a chain-to-chain RMSD over all common Cα atoms of 1.8 Å. FabH has no close human homolog, so the availability of structures from multiple orthologs may be useful in designing antimicrobial drugs with cross-species reactivity.

**Figure 2:**
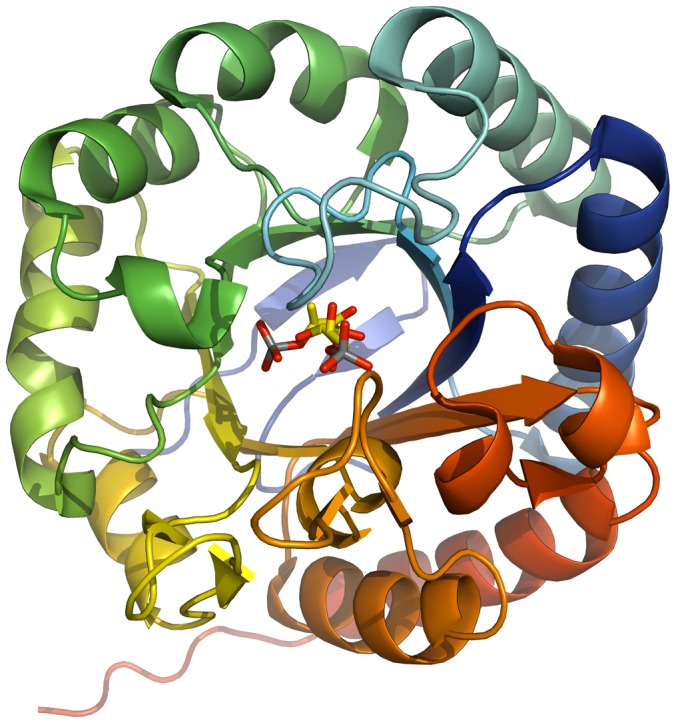
KDOP synthase from *B. pseudomallei*. KDOP synthase (2-dehydro-3-deoxyphosphooctonate aldolase, BURPS1710b_3264, PDB: 3UND with bound D-arabinose-5-phosphate), a KDO2-lipid A biosynthesis enzyme with a TIM barrel structure, was one of five structures solved for orthologs of the putative essential *B. thailandensis* gene, Bth_I1893.

KDOP synthases are involved in KDO2-lipid A or lipopolysaccharide biosynthesis, and catalyze the conversion of phosphoenolpyruvate and D-arabinose 5-phosphate to 2-dehydro-3-deoxy-D-octonate 8-phosphate [33]. KDOP synthase (2-dehydro-3-deoxyphosphooctonate aldolase) has no close human homolog, and we found the *B. thailandensis* gene, BTH_I1893, to be essential. We solved structures for five orthologs of this gene: from *B. ambifaria* (BamMC406_2018, PDB: 3T4C), *B. cenocepacia* (BCAL2180, PDB: 3TML, with bound sulfate), and *B. pseudomallei* (BURPS1710b_3264, PDB: 3SZ8, 3UND, and 3TMQ). Figure 2 shows the TIM barrel structure of the enzyme. Again, the orthologs have a high degree of structural similarity, with overall Cα RMSD values of 1.2 Å for 3UND and 3TML and 1.8 Å for 3UND and 3T4C.

**Figure 3:**
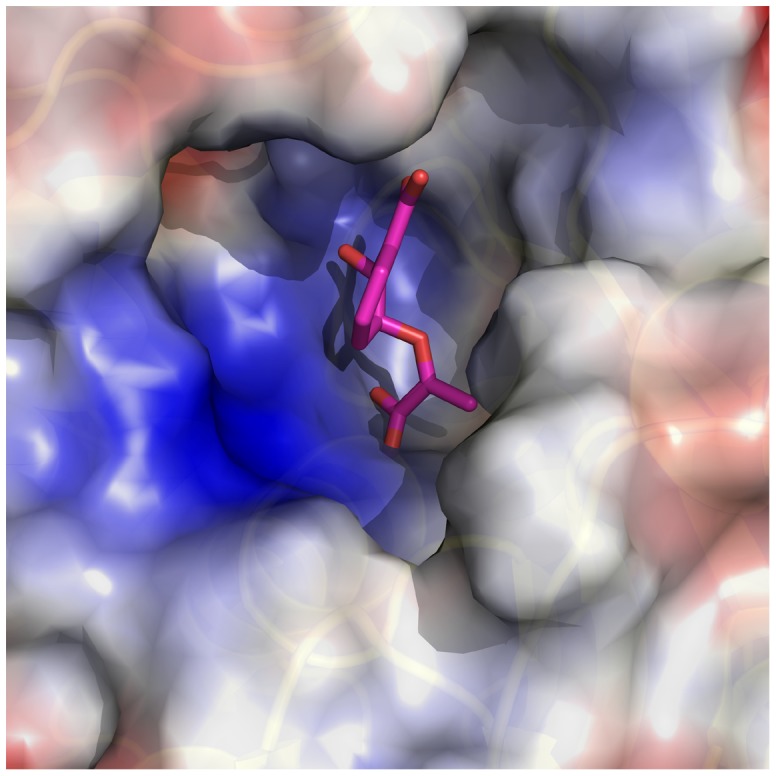
Isochorismatase from *B. thailandensis*. The isochorismatase family protein (BTH_II2229, PDB: 3TXY) from *B. thailandensis*, is shown in electrostatics surface representation with bound isochorismate taken from the *P. aeruginosa* ischorismatase, PhzD (PDB: 1NF8). 3TXY and 1NF8 have 30% sequence identity and an overall Cα RMSD of 1.7 Å. By aligning 1NF8 and 3TXY, the active site of 3TXY can be identified as a large pocket with a combination of hydrophobic (white) and positively charged (blue) amino acid residues.

Isochorismate is an intermediate in the synthesis of siderophores such as enterobactin and vibriobactin, which are crucial for microorganisms to acquire iron from their surroundings [34], [35]. We solved a structure for the putative isochorismatase family protein, BTH_II2229 (PDB: 3TXY) from *B. thailandensis*. This protein has no close human homolog, but shows sequence and structural similarity to PhzD from *P. aeruginosa* (PDB: 1NF8, 30% sequence identity, 47% coverage, 1.7 Å overall Cα RMSD) (Figure 3). PhzD catalyzes an intermediate reaction in the formation of phenazine-1-carboxylic acid (PCA). Derivatives of PCA are virulence factors and natural antibiotics in several pathogenic strains of bacteria, including *Pseudomonas* and *Streptomyces* [36]. This structure may be useful in selecting compounds to validate isochorismatase as a drug target in *Burkholderia* and other GNRs.

**Figure 4:**
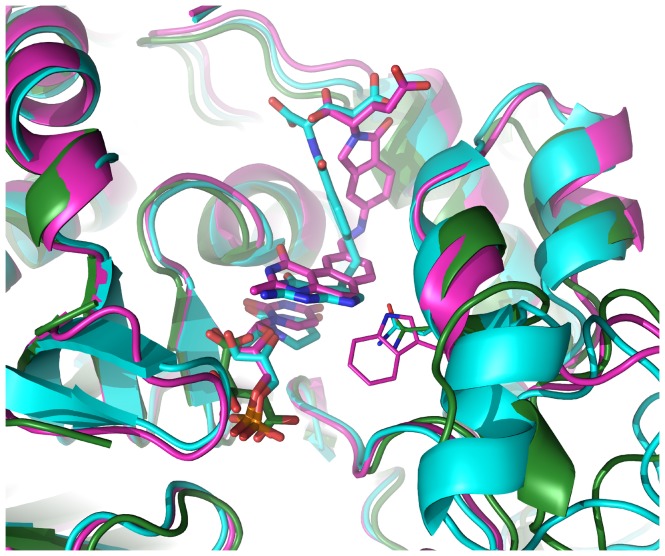
Thymidylate synthase (TS) from *B. thailandensis*, *E. coli* and *Homo sapiens*. TS from human (cyan, PDB: 1SYN) and *E. coli* (magenta, PDB: 1JU6) show similar active site structure as TS from *B. thailandensis* (green, PDB: 3V8H, C-terminal residues removed for clarity). A canonical active site tryptophan (W83 in *E. coli*) for bacterial sequences is replaced in *B. thailandensis* by asparagine, the residue observed in this position in human TS (side chains shown in stick representation, below and to the right of the bound ligand, citric acid).

Thymidylate synthase (TS) is a proven anti-cancer drug target with active ongoing research for its potential as an antibacterial [37]–[41]. The high sequence and structural homology across TS enzymes from human and many parasite species, particularly within active site residues, creates a challenge for obtaining drug selectivity [42], [43]. The *B. thailandensis* TS protein (BTH_I1680, PDB: 3V8H) has an arginine residue substituted for a canonical active site tryptophan (W83 in *E. coli*); arginine is also the side chain found in human TS (Figure 4). While, the difference in amino acid identity in the active site between human and *Burkholderia* proteins may be too small to develop a broad-spectrum antibiotic capable of host-parasite selectivity, large subdomain differences between TS enzymes from different species (not shown) may provide an alternate drug development strategy. An additional strategy in targeting TS is to simultaneously target thymidine kinase (TK), since bacteria may circumvent TS inhibition through TK activity [44]. In this regard, we have also solved a structure for TK in *B. thailandensis* (BTH_I2154, PDB: 3V9P). A therapy targeting both TS and TK enzymes could prolong the lifespan of inhibitors with human-parasite selectivity.

**Figure 5:**
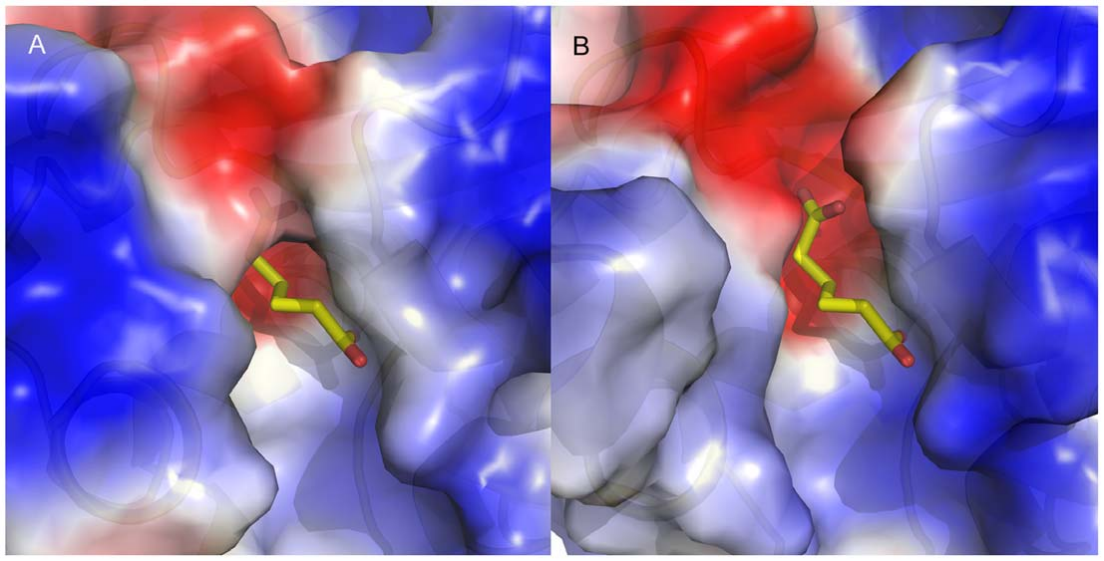
Peptidyl-tRNA hydrolase from *B. thailandensis*. (A) The electrostatic surface of unliganded peptidyl-tRNA hydrolase (PTH, Bth_I0472, PDB: 3V2I) from *B. thailandensis* is superimposed with a cartoon representation of a structure from *P. aeruginosa* with bound adipic acid (PDB: 4DHW). The channel in unliganded 3V2I is closed due to adjacent flexible loops. (B) The electrostatics surface of 4DHW reveals an open, charged channel. 3V2I and 4DHW have 44% sequence identity and a similar overall fold (2.0 Å RMSD over all common Cα atoms). Discovery of a ligand that binds the alternately charged channel (positive/negative/positive) could block the reaction and prevent protein synthesis.

Peptidyl-tRNA hydrolase (PTH) is an enzyme that cleaves the ester bond on peptidyl-tRNAs that are stalled on the ribosome, releasing an *N*-substituted amino acid and free tRNA [45]. Inhibition of PTH depletes the supply of aminoacyl-tRNA, stopping protein synthesis. We identified PTH as essential in *B. thailandensis*, and it has been identified previously as essential in other bacteria [46], [47]. The structure for PTH in *B. thailandensis* (BTH_I0472, PDB: 3V2I) has a large, charged binding pocket (Figure 5). Discovery of a ligand that binds the alternately charged (positive/negative/positive) channel could block the reaction and prevent protein synthesis. PTH has a human homolog (Q86Y79 UniProtKB AC, no PDB structure available) with 36% sequence identity and 87% coverage, so further structural comparison using a 3D model of the human protein would be necessary to determine whether drug selectivity is possible. However, achieving selectivity may not be necessary since eukaryotes possess multiple PTH activities [48].

## Discussion

Here we report a functional and structural genomics effort that applied saturation-level transposon mutagenesis and next generation sequencing (Tn-seq) to identify essential genes in *B. thailandensis*, followed by high-throughput structure determination. We used an “ortholog rescue” approach to maximize structural coverage of these gene families, which are likely to be essential not only in *B. thailandensis,* but also in related, but more virulent, *Burkholderia* species, such as *B. pseudomallei*. A large fraction of the genes (83%, 336/406) that we identified have homologs previously identified as essential either in *B. cenocepacia* [11], in *P. aeruginosa* [16], or in other prokaryotes listed in the Database of Essential Genes [15]. Of the remaining 70, some are likely to be essential but have not been identified previously, as there had been no experimental genome-wide essentiality studies in *Burkholderia* prior to this study. A small percentage of our putative essential genes may be false positives – genes wrongly identified as essential. These are most likely to be small genes which due to their size are most likely to have eluded mutagenesis, or genes with close to the threshold of three insertions per kB in the 5–90% portion of the ORF (in two independent mutant pools) (Table S1). This threshold was chosen based on a survey of genes thought to be essential based on annotated function, in which small numbers of insertions were detected, and was used to reduce false negatives; for example, rare insertions in transiently duplicated genes or within intra-domain regions may not fully abrogate essential function. False negatives are still possible, and are most likely to be genes that possess nonessential domains tolerant of transposon insertions.

The number of essential genes identified, 406, falls within the range of values estimated for other bacteria using experimental approaches such as genome-wide gene disruption or mutagenesis [10], [12], [13]. Experimentally determined estimates of the number of essential genes in pathogenic bacteria range from <200 to >600. By comparing the genomes of all 51 species in the order *Burkholderiales* and clustering using OrthoMCL, Juhas *et al.* identified 610 ortholog groups conserved among all 51 species (the “core genome”), corresponding to 649 genes in *B. cenocepacia* [11]. Of these 649 genes, 454 had homologs in the Database of Essential Genes (DEG). However, both computational gene conservation analysis and experimental methods that use lower mutation rates per gene (upon which much of the DEG is based) are likely to overestimate the number of essential genes.

By using an ortholog rescue strategy for insoluble or difficult to crystallize targets, we increased our structural coverage of *B. thailandensis* essential genes from 31/406 (7.6%) to 49/406 (12.1%) (Table 3, Table S2). Such an approach has been used previously in high-throughput structure determination efforts to similarly improve the overall gene-to-structure efficiency for closely related protein sequences. In *Plasmodium*, the ortholog rescue approach was able to improve the protein solubility rate to 229/468 target genes (49%) resulting in 32 structures (6.8%) [18]. SSGCID has also improved the gene-to-structure rate from 11% for *Mycobacterium tuberculosis* targets to 36% by using orthologs from nine other *Mycobacterium* species [manuscript in preparation]. However, the underlying rationale for this approach – that ortholog structures are sufficiently similar to serve as surrogates in drug design – has rarely been verified with experimental data. For the seven pairs of ortholog structures (with no bound ligand) solved in this study, the average overall Cα RMSD was 1.5±0.5 Å (Table S4), indicating a high degree of structural similarity. This structural similarity suggests that the ortholog approach is an efficient method to obtain useable structures from otherwise intractable targets, thereby lowering the barrier to structure-based drug design targeting infectious organisms. Ortholog structures may also be useful in designing broad-spectrum antibiotics with cross-species activity, and by representing a variety of functionally conservative point mutations in the active site may be useful in developing drugs less susceptible to mutations that cause drug resistance.

Of the 56 *Burkholderia* protein targets with a structure solved, 25 possess properties of a potential antimicrobial drug target: *i.e.*, they were experimentally identified as an essential gene product or are a close ortholog; they are members of a metabolic pathway containing at least two essential enzymes (as listed in the DEG); they possess a deep, druggable pocket large enough to envelop a compound of at least six non-hydrogen atoms; and they lack a close human homolog, reducing the chance of host toxicity. Thus we have solved structures for 25 *Burkholderia* proteins that appear worthy of further validation as drug targets, including chemical validation to determine whether blocking the target affects cell growth and viability *in vivo*.

### Conclusions

We have combined an experimental genome-wide essentiality screen in *B. thailandensis*, using a high rate of insertions per gene, with high-throughput structure determination and an ortholog rescue approach to achieve a significant structural coverage of essential genes. Using only seven *Burkholderia* species to select orthologs of essential genes, we solved structures for 49/406 essential gene families, and for 56 total *Burkholderia* protein targets (including seven ortholog replicates). Of these 56 targets, 25 satisfied criteria for being a potential antimicrobial drug target. By increasing the number of species used to select orthologs, future efforts may come closer to complete coverage of the essential structomes of other infectious organisms. The resulting collection of structures and information about target essentiality and solubility provides a resource for development of new antibiotics to treat *Burkholderia*-related infectious diseases.

Expression clones and proteins created in this study can be freely obtained *via* BEI Resources (http://www.beiresources.org/StructuralGenomicsCenters.aspx) and through the SSGCID website (http://www.ssgcid.org/home/index.asp). Clones and proteins may be searched for using the SSGCID Target IDs listed in Table S2.

## Materials and Methods

### Experimental Identification of Essential Genes

*B. thailandensis* strain E264 (ATCC 700388) was mutagenized with transposon T23 (ISlacZ_p_rhaBout_-Tp/FRT) by conjugal delivery from *E. coli* strain SM10/λpir of suicide plasmid pLG99, which bears the transposon and the transposase gene. Insertion mutants were selected by incubation for 24 h at 37°C on TYE agar (10 g tryptone, 5 g yeast extract, 8 g sodium chloride and 15 g agar per L) supplemented with 50 µg/mL trimethoprim (to select for insertion mutants) and 100 µg/mL streptomycin (to select against the *E. coli* donor). Mutants were pooled by scraping cells off the selective media, and DNA from the pools purified by DNeasy Blood & Tissue Kit (Qiagen). Tn-seq analysis of the pooled DNA was carried out as described [25] using oligonucleotides specific for transposon T23 (sequences available upon request). Two independent pools were generated and analyzed (Table 1). The number of chaste sequence reads obtained for the two pools were 26,398,169 and 11,888,155, of which 24,020,048 and 10,001,776, respectively, mapped to the E264 genome. Since insertions near gene termini may not represent null mutations, insertions within the 3′ 5% or 5′ 10% of each ORF were ignored when assessing essentiality. Additionally, since rare insertions in transiently duplicated genes or within intra-domain regions may not fully abrogate essential functions, genes with fewer than three insertions per kB (in the 5–90% portion of the ORF) were also included in the analysis. The limit of three insertions per kB was determined based on a survey of putatively essential genes (by annotated gene function) in which small numbers of insertions were detected. Thus, for a gene to be assigned as (putatively) “essential”, it needed to receive fewer than three hits per kB in the 5–90% region in both mutant pools.

### Bioinformatics

Genomes for all *Burkholderia* species were downloaded from the Wellcome Trust Sanger Institute website (http://www.sanger.ac.uk/resources/downloads/bacteria/) or from the *Burkholderia* Genome Database (http://www.burkholderia.com/download.jsp). Sequences of previously determined essential genes were obtained from the UniProtKB website (http://www.uniprot.org) and from the Database of Essential Genes (http://tubic.tju.edu.cn/deg/) [15]. BlastP searches were performed using Geneious software (Biomatters; www.geneious.com), using default settings with an *E*-value cutoff of 1×10^−10^ and minimum sequence identity and coverage of 40% and 70%, respectively, for selecting orthologs, and 30% and 50%, respectively, for identifying human homologs and homologs among genes identified previously as essential. For selecting orthologs, sequences identified by BlastP search were clustered into ortholog groups using OrthoMCL [27], [28]. E.C. numbers and metabolic pathway information was obtained from the KEGG [26]. RMSD calculations were performed using Dali (http://ekhidna.biocenter.helsinki.fi/dali_server/start) [49].

### High-throughput Protein Expression, Purification, Crystallization, and Structure Determination

PCR, cloning, screening, sequencing, expression screening, scale-up, and purification of proteins were performed as described previously [50], [51]. DNA templates for PCR amplification were obtained from Joe Mongous (University of Washington, Seattle) for *B. thailandensis* E264 and *B. ambifaria* MC40-6, from Jane Burns (Seattle Children’s Pediatrics) for *B. cenocepacia* J2315 and *B. multivorans* ATCC 17616, from Mary Lidstrom (University of Washington) for *B. phymatum* STM815 and *B. xenovorans* LB400, from Eshwar Mahenthiralingam (Cardiff University, UK) for *B. vietnamiensis* G4, and from American Type Tissue Culture for *B. pseudomallei* 1710b. Crystal trials, diffraction, and structure solution were performed as described previously [52], [53].

## Supporting Information

Table S1 **Putative essential genes in** ***B. thailandensis*** **E264.**
(XLSX)

Table S2 ***Burkholderia*** **protein structures (expanded version).**
(XLSX)

Table S3 **Structural characteristics of proteins reported.**
(XLSX)

Table S4 **Comparison of ortholog structures.**
(XLSX)
